# The association of different dimensions of anhedonia in the relationship between depressive symptoms and self-harm in adolescents with mood disorders

**DOI:** 10.3389/fpsyt.2026.1845853

**Published:** 2026-06-04

**Authors:** Jiping Xiao, Dandan Liu, Li Cheng, Mian Zhang, Ming Wu, Nan Du, Li Zhu, Daomin Zhu

**Affiliations:** 1 Affiliated Psychological Hospital of Anhui Medical University, Hefei, China; 2 Anhui Mental Health Center, Hefei, China; 3 Department of Child and Adolescent Psychiatry, Hefei Fourth People's Hospital, Hefei, China; 4 Department of Sleep Disorders, Hefei Fourth People's Hospital, Hefei, China

**Keywords:** adolescents, anhedonia, mood disorders (MD), non-suicidal self-injury (NSSI), self-harm, suicide attempt (SA), suicide ideation

## Abstract

**Objective:**

An increasing number of adolescents with mood disorders engage in self-harm. Anhedonia is independently associated with self-harm. Exploratory analyses in this cross-sectional study examined the associations of different dimensions of anhedonia in self-harm to provide additional theoretical support for self-harm intervention.

**Methods:**

This study recruited 252 adolescents aged 12–18 years with mood disorders, and divided them into high-risk (n=143) and low-risk (n=109) groups based on suicide risk, and recruited 120 normal control participants. Data collected included depressive and anxiety symptoms (HAMD-24, HAMA-14), different dimensions of anhedonia, including the anticipatory anhedonia (TEPS-A)and consummatory anhedonia (TEPS-C), Social Anhedonia (RSAS), and the somatic Anhedonia (RPAS), and suicidal ideation (PANSI), non-suicidal self-injury (NSSI), and suicide attempts (SA).

**Results:**

All anhedonia dimensions and self-harm indicators differed significantly across groups (high-risk > low-risk > controls, all P < 0.001). anticipatory anhedonia (TEPS-A) [B = –0.194, P = 0.043, 95% CI (–0.382, –0.006)] and consummatory anhedonia (TEPS-C) [B = –0.228, P = 0.016, 95% CI (–0.413, –0.042)] were independently and negatively associated with PANSI. Somatic anhedonia (RPAS) were independently and positively associated with NSSI [B = 0.642, P = 0.001, 95% CI (0.255, 1.030)] and SA (OR = 1.050, P = 0.024, 95% CI [1.006, 1.097]). Anticipatory anhedonia (TEPS-A) showed a significant indirect effect in the relationship between depressive symptoms and PANSI [B = 0.051, 95% CI (0.006, 0.105)]. Somatic anhedonia (RPAS) showed a significant indirect effect in the relationship between depressive symptoms and NSSI [B = 0.241, 95% CI (0.118, 0.389)], and together with NSSI and PANSI, formed a significant sequential indirect effect to SA [B = 0.0012, 95% CI (0.0002, 0.0026)].

**Conclusion:**

Different anhedonia dimensions demonstrate distinct associations with self-harm: anticipatory anhedonia was associated with suicidal ideation, whereas somatic anhedonia connects to NSSI and suicide attempts. These findings support dimensional-specific clinical assessment and targeted interventions. Causal inference is not possible due to the cross-sectional design, and longitudinal studies are needed to establish temporal relationships and causal mechanisms.

## 1 Introduction

Self-harm is a general term for intentional acts of self-injury, encompassing both suicide attempt (SA) and non-suicidal self-injury (NSSI). It is a pathological behavior characterized by complex mechanisms and extremely serious consequences. Suicide attempt (SA) and non-suicidal self-injury (NSSI) are distinct yet inextricably linked, and both can serve as predictors of suicide (1, 2). In the past decades, an increase in the incident rate of suicide attempt (SA) and NSSI in adolescents has been observed, which has become a public health concern affecting adolescents worldwide (3). Adolescence is a period of high prevalence of mental health problems, with mood disorders (MD), such as depression and bipolar disorder, showing significantly increased prevalence in this population. Mood disorders are high-risk populations for NSSI and suicidal behavior. The suicide attempt rate among adolescents with bipolar disorder (BD) is 31.5%, for major depressive disorder (MDD) it is 20.5%, and for pure mania/hypomania episodes it is 8.5% (4). Adolescents with bipolar disorder engage in self-harm more frequently and suffer worse consequences than those with depressive episodes (5). The risk of suicide death in patients with bipolar disorder is 60 times that of the general population (6). The lifetime prevalence of suicide attempts in adolescents with mood disorders is much higher than in healthy individuals (7). In multicenter studies in China (8), the detection rate of NSSI in patients with bipolar disorder has significantly increased and is closely associated with suicidal ideation. A meta-analysis suggests that the rate of NSSI among Chinese adolescents with depression reaches 41.4%-66.7% (9).

Anhedonia refers to the loss of interest or pleasure in all or almost all activities, manifested as an inability to experience joy or a loss of interest in things that were previously enjoyable (10). In recent years, research has found that the anhedonia is associated with various mental illnesses, especially depressive disorders bipolar disorder and schizophrenia (11, 12). Anhedonia also affects the prognosis and outcomes of mental illnesses. It not only predicts the response to combined treatments but also predicts poorer treatment outcomes (13). McMakin’s research (14) found that anhedonia can serve as an early marker for identifying adolescents with poor prognosis. Traditionally, anhedonia has been viewed as a unidimensional construct, but recent research has revealed that it has a complex dimensional structure (15). From a temporal perspective, it can be divided into anticipatory anhedonia (a decreased ability to anticipate pleasure from future rewarding events) and consummatory anhedonia (a decreased ability to experience pleasure from current activities) (16). From a content perspective, it can be divided into somatic anhedonia (a reduced pleasure from bodily sensory stimuli) and social anhedonia (a decreased ability to experience pleasure in social activities) (17). In recent years, the relationship between anhedonia and self-injury and suicide has always been one of the research hotspots, but the results have been inconsistent. Loas (18) found in their study of hospitalized patients with mood disorders that anticipatory anhedonia was associated with suicidal ideation, whereas consummatory anhedonia was not. However, a subsequent study (2019) (19) found that the loss of pleasure (consummatory component), rather than the loss of interest (anticipatory component), was a significant predictor of suicidal ideation among medical students. Winer (20) suggested that social anhedonia (loss of interest in people) predicts suicide risk better than somatic anhedonia, as it reflects social withdrawal and interpersonal isolation. Anhedonia is significantly associated with a history of NSSI and is related to interpersonal functioning in NSSI (21). Loas (10) believe that the social specificity of anhedonia may be overemphasized, and general dissatisfaction is more predictive. Despite growing recognition that anhedonia is a transdiagnostic risk factor for self-harm, several critical gaps remain exist. First, existing studies have yielded inconsistent findings regarding which specific dimension of anhedonia most strongly predicts suicidal ideation versus NSSI or suicide attempts. These discrepancies likely stem from methodological heterogeneity—particularly the use of unidimensional or dimensionally nonspecific assessment tools, and the inclusion of mixed samples spanning nonclinical populations, and diverse diagnostic categories. Second, whether these dimensional-specific associations operate independently of, or in conjunction with, depressive symptom severity remains unclear, as few studies have systematically examined the mediating roles of different anhedonia dimensions within the depression–self-harm continuum.

Accordingly, this study aimed to: (1) explore the associations of different dimensions of anhedonia with suicidal ideation, NSSI, and suicide attempts in a clinically homogeneous sample of adolescent inpatients with mood disorders; (2) examine the distinctions and potential pathways of anhedonia between depressive symptoms and different self-harm outcomes. By employing dimension-specific assessment tools and distinguishing among self-harm outcomes, this study conducted some theoretical exploration for developing targeted prevention and intervention strategies for this high-risk population.

## 2 Methods

### 2.1 Participants

patients group: A total of 252 adolescent inpatients diagnosed with mood disorders according to ICD-10 were recruited from Hefei Fourth People’s Hospital between January 2023 to June 2025. Diagnoses were confirmed by two psychiatrists independently using the Mini International Neuropsychiatric Interview (MINI) for ICD-10 diagnostic criteria. Discrepancies were resolved through consensus discussion with a senior psychiatrist. Based on the assessed suicide risk, participants were classified into low‑risk and high‑risk groups. The main assessments of suicide risk (HAMD item 3 was used to assess suicide risk (22)), include: 0=absent, 1=feels life is not worth living, 2=any thoughts of possible death of herself/himself, 3=suicide ideation/ suicide gesture, and 4=attempts at suicide. Cases with scores 0-2 are included in the low-risk group, while 3/4 are included in the high-risk group. Inclusion criteria comprised: (1) Meeting the diagnostic criteria (ICD-10) for mood disorders,mainly including major depressive disorder, bipolar depressive episodes, bipolar mixed episodes. (2) Age 12–18 years. (3) Depressive symptoms are the prominent symptom at the moment, with a score of ≥20 on the 24-item Hamilton Depression Scale (HAMD-24). (4) Ability to complete assessments independently or with researcher assistance. Exclusion criteria comprised: (1) Organic brain disease or severe physical illness; (2) Comorbidity with other mental disorders; (3) A documented history of drug or alcohol abuse.

Healthy control group: 120 healthy adolescents were recruited from a public middle school in Hefei (matched for socioeconomic catchment area with the hospital) during a single recruitment wave (March–April 2024) to avoid temporal confounding.Stratified random sampling was conducted based on grade level (grades 7–12) and sex to match the distribution of the patient group. Propensity score matching was not employed given the small sample size and limited covariates. Exclusion criteria comprised: (1) Organic brain disease or severe physical illness; (2) Current or past diagnosis of any mental disorder; (3) history of self-harm or suicidal behavior; (4) Score ≥20 on the 24-item Hamilton Depression Scale (HAMD-24). All participants and their legal guardians provided written informed consent. The control group did not differ significantly from the patient group in age, sex, or education level (all P > 0.05).

The research process is as follows: Upon admission, patients are clearly diagnosed by two psychiatrists and classified into high-risk and low-risk groups based on their suicide risk, and then demographic data and clinical scale assessments are completed within three days of admission.

### 2.2 Materials

General data and clinical scale assessment: Relevant assessments were completed within three days of admission, including a general demographic survey (covering gender, age, education level, and for the case group also: disease duration, whether it is first onset or relapse, current medication) and related scale assessments. In this study, the 24-item Hamilton Depression Scale (HAMD-24) and the 14-item Hamilton Anxiety Scale (HAMA-14) were used to evaluate depressive and anxiety symptoms.

#### 2.2.1 Temporal experience of pleasure scale

The state of anticipatory anhedonia and consummatory anhedonia were assessed by Chinese version of TEPS (Temporal Experience of Pleasure Scale) (23), which added a “context” dimension to the original version based on the Chinese culture. Chinese version of TEPS consisted of 20 items and covers four dimensions: abstract anticipatory, contextual anticipatory, abstract consummatory and contextual anticipatory. A 6-point Likert scale was used, ranging from 1 (very false for me) to 6 (very true for me), Since we only focused on the differences between consummatory anhedonia and anticipatory anhedonia, we summed the abstract and contextual scores of the same categories to get the consummatory (TEPS-C) score and anticipatory (TEPS-A) score. A lower score indicates more severe anhedonia.

#### 2.2.2 Revised physical anhedonia scale and revised social anhedonia scales

The state of somatic anhedonia and social anhedonia were assessed by Chinese version of RPAS (Revised physical anhedonia scale), and Chinese version of RSAS (Revised social anhedonia scale) which were developed by Chapman (24) based on Rado and Meehl’s theory. RPAS contained 61 items assessing the degree of pleasure experienced in physical aspects. RSAS contained 40 items assessing the degree of anhedonia in social interpersonal interactions. Items are answered “yes” or “no,” with “yes” scored as 1 and “no” as 0; some items are reverse-scored. The total score is the sum of all items. A hicher score indicates more severe anhedonia.Both the original English RPAS/RSAS (α=0.74/0.85) and the Chinese RPAS/RSAS (Cronbach’s α=0.75/0.94) have good internal consistencies.

#### 2.2.3 Positive and negative suicide ideation scale

The state of suicide ideation were assessed by Chinese version of PANSI (Positive and Negative Suicide Ideation Scale) (25). This scale is a widely used self-report questionnaire developed by Osman and colleagues, which is designed to evaluate suicidal ideation in adolescents. It contained 14 items across 2 dimensions (positive and negative suicide ideation), rated on a 5-point scale. Positive items are reverse-scored, and negative items are positively scored. Higher scores indicate higher levels of suicidal ideation. Both the two dimensions, negative/positive(Cronbach’s α=0.95/0.81), and total scale(Cronbach’s α=0.92) have good internal consistencies.

#### 2.2.4 Revised the adolescent self-harm behavior questionnaire (NSSI)

The state of non-suicidal self-injury were assessed by Revised the adolescent self-harm behavior questionnaire (26). Feng Yu further revised it based on Gratz’s (27) and Zheng’s (28) work. This scale contained 36 items examining adolescent self-injurious behavior through two parts: frequency of self-injurious behavior and average degree of physical harm. Self-injury frequency is scored on a 4-point Likert scale: 0 (0 times), 1 (1 time), 2 (2–4 times), and 3 (5 times or more). Degree of physical harm is assessed on a 4-point Likert scale from 0 (none) to 4 (extremely severe). If the frequency is “0”, the degree of harm does not need to be filled in. The total self-injury score is the sum of the products of past self-injury frequency and degree of physical harm; higher scores indicate more severe self-injurious behavior. The Cronbach’s α coefficient of this scale was 0.85.

### 2.3 Statistical analysis

Statistical analysis was performed using SPSS 26.0. The analytic sequence proceeded as follows: (1)Between-group Comparisons employed Chi-square tests for categorical variables and one Way ANOVA for continuous variables. *post-hoc* pairwise comparisons and correction using the bonferroni method were conducted; Independent samples t-test was used to compare differences in disease duration between patient groups; (2) Spearman’s correlation was used to explore associations among key variables; To control for inflated familywise Type I error across 36 pairwise comparisons, the Bonferroni correction was applied, yielding a corrected significance threshold of α = 0.00139 (0.05/36). Correlations with uncorrected P < 0.00139 are reported as significant; less stringent associations are noted as trends. (3) Hierarchical multiple regression analyses were performed to examine independent associations; All regression analyses were conducted using hierarchical multiple regression with the ENTER method at each block. In Block 1, demographic and clinical covariates were entered to control for potential confounding effects. In Block 2, the core variables of interest were entered: four dimensions of anhedonia (TEPS-A, TEPS-C, RPAS, RSAS), suicidal ideation (PANSI), and non-suicidal self-injury (NSSI), depending on the specific model. Diagnostic subtype and HAMD were both included despite conceptual overlap to account for diagnostic heterogeneity within the mood disorder sample and to isolate variance specific to symptom severity. The simultaneous inclusion of PANSI and NSSI as predictors should be interpreted with caution given their conceptual overlap and shared variance. (4) Finally, to examine statistical patterns consistent with hypothesized psychological mechanisms linking depressive symptoms and self-harm, the PROCESS plugin was used to construct a exploratory mediation models.The indirect effects model was estimated using maximum likelihood estimation and tested using the Bootstrap method with 5,000 bootstrap repetitions. The significance of path effects was determined based on whether the 95% confidence interval from the Bootstrap analysis included zero.Given the cross-sectional design, significant indirect effects are interpreted as statistical association patterns consistent with theoretical models, not as evidence of causal mechanisms or temporal sequencing.

## 3 Results

### 3.1 General demographic characteristics results and divergent results

Regarding general demographics, there were no significant differences among the three groups in age, gender, or education level (all *P* > 0.05). In terms of diagnostic types in the patient group, in the low-risk group, there were 82 cases of depression, 15 cases of bipolar mixed states, and 12 cases of bipolar depression; in the high-risk group, there were 128 cases of depression, 11 cases of bipolar mixed states, and only 5 cases of bipolar depression. The distribution across suicide risk groups differed significantly (χ² = 9.545, *P* = 0.008). *Post-hoc* pairwise comparisons and correction using the Bonferroni suggested the high-risk group had a higher proportion of depression (89.5% vs. 75.2%) and lower proportion of bipolar depression (3.5% vs. 11.0%) compared to the low-risk group. low-risk and high-risk groups have no significant statistical difference in disease duration percentage of first episode. The suicide attempt rate in the high-risk group was significantly higher than that in the low-risk group (*χ^2^* = 252.000, *P* < 0.001). Regarding anhedonia, there were significant differences among the three groups in the TEPS (including TEPS-A and TEPS-C),RPAS, RSAS, PANSI, NSSI. (all *P* < 0.001). (Details are shown in **Table 1**).

**Table 1:** Demographic and clinical information of study subjects ( $\bar{\text{x}}$ ± s/n).

Variables	Patient group		Healthy control group ③ (n=120)	χ^2^/*t/F*	Comparison between groups
	Low risk group① (n=109)	High risk group② (n=143)			
gender				2.909	
Female	76	116	80		
Male	33	37	40		
first episode				3.561	
no	54(49.5%)	89(62.2%)			
yes	55(50.5%)	54(37.8%)			
diagnosis type				9.545^**^	
depression	82(75.2%)	128(89.5%)			
bipolar depression	12(11.0%)	5(3.5%)			
bipolar mixed states	15(13.8%)	10(7.0%)			
SA				252.000	
no	82	75			
yes	27	68			
Age/years	15.40 ± 1.66	15.16 ± 1.58	15.18 ± 1.62	0.801	
Education/years	9.66 ± 1.62	9.50 ± 1.53	9.53 ± 1.66	0.355	
Disease duration/months	59.58 ± 59.98	60.68 ± 67.12		1.562	
HAMA	18.36 ± 6.80	22.87 ± 6.78	7.08 ± 4.03	230.763^***^	②>①>③
HAMD	26.55 ± 7.39	38.99 ± 7.51	5.88 ± 3.28	876.743^***^	②>①>③
RSAS	20.69 ± 7.38	24.11 ± 7.09	12.77 ± 5.42	96.519^***^	②>①>③
RPAS	25.73 ± 12.89	32.67 ± 10.04	13.48 ± 5.83	123.236^***^	②>①>③
TEPS	74.09 ± 18.13	60.90 ± 16.09	85.61 ± 15.77	72.390^***^	③>①>②
TEPS-A	36.00 ± 9.73	28.25 ± 8.99	42.70 ± 7.99	86.216^***^	③>①>②
TEPS-C	37.05 ± 9.92	30.87 ± 9.52	42.74 ± 8.88	51.702^***^	③>①>②
PANSI	41.60 ± 10.30	46.57 ± 10.40	25.03 ± 9.79	151.640^***^	②>①>③
NSSI	20.78 ± 22.40	32.09 ± 28.48	3.77 ± 9.50	53.721^***^	②>①>③

① indicates low-risk group, ② indicates high-risk group, and ③ indicates healthy control group. HAMD, Hamilton Depression Rating Scale; HAMA, Hamilton Anxiety Scale; TEPS, Temporal Experience of Pleasure Scale; TEPS-C, consummatory score and TEPS-A, anticipatory score. RPAS, Revised somatic anhedonia scale; RSAS, Revised social Anhedonia scales; PANSI, Positive and Negative Suicide Ideation Scale; NSSI, Revised the adolescent self-harm behavior questionnaire. *statistically significant at p<0.05 level; **statistically significant at p<0.01 level; ***statistically significant at p<0.001 level.

### 3.2 Correlation analysis

HAMD were negatively correlated with the TEPS and its two-dimensions (TEPS-A and TEPS-C). HAMD were positively correlated with RPAS, RSAS, PANSI, NSSI. These findings suggest that depressive symptoms are associated with various dimensions of anhedonia, suicidal ideation, and non-suicidal self-injury. Meanwhile there is a significant correlation between TEPS (including the TEPS-A and TEPS-C dimensions), RPAS, RSAS, PANSI, and NSSI; furthermore, the severity of anhedonia across different dimensions is associated with the severity of suicidal ideation and non-suicidal self-injury. (Details are shown in **Table 2**).

**Table 2:** Correlation analysis of HAMD, HAMA, TEPS (TEPS-A,TEPS-C), RPAS, RSAS, PANSI, and NSSI.

Variables	HAMD	HAMA	TEPS	TEPS-A	TEPS-C	RSAS	RPAS	PANSI
HAMD	1							
HAMA	0.569^***^	1						
TEPS	-0.194^***^	-0.025	1					
TEPS-A	-0.214^***^	-0.073	0.883^***^	1				
TEPS-C	-0.172^***^	0.018	0.934^***^	0.709^***^	1			
RSAS	0.224^***^	0.083	-0.561^***^	-0.542^***^	-0.497^***^	1		
RPAS	0.230^***^	0.094	-0.679^***^	-0.628^***^	-0.642^***^	0.583^***^	1	
PANSI	0.221^***^	0.182	-0.372^***^	-0.336^***^	-0.346^***^	0.251^***^	0.271^***^	1
NSSI	0.179^***^	0.115	-0.338^***^	-0.312^***^	-0.292^***^	0.325^***^	0.399^***^	0.339^***^

*statistically significant at p<0.0014(0.05/36) level; **statistically significant at p<0.0003(0.01/36) level; ***statistically significant at p<0.00003(0.001/36) level.

### 3.3 Regression analysis results

All regression analyses were conducted using hierarchical multiple regression with the ENTER method at each block. Based on the above differences between groups and the results of correlation analysis, we further controlled for diagnosis type and anxiety symptoms as confounding factors.

Associations with Suicidal Ideation (PANSI): A hierarchical multiple linear regression was conducted with PANSI as the dependent variable. In Block 1, diagnosis subtype, and anxiety symptoms (HAMA) were entered as covariates. In Block 2, depressive symptoms (HAMD), four dimensions of anhedonia (TEPS-A, TEPS-C, RSAS, RPAS), and Non-Suicidal Self-Injury (NSSI) were simultaneously entered. Block 1 accounted for 4.4% of the variance (R² = 0.044, adjusted R² = 0.032, F[3, 239] = 3.635, P = 0.014). The addition of Block 2 significantly improved the model, explaining an additional 18.7% of variance (ΔR² = 0.187, ΔF[6, 233] = 9.457, P < 0.001). In the full model, after controlling for diagnosis subtype, and anxiety symptoms, anticipatory anhedonia (TEPS-A) [B = –0.194, SE = 0.095, β = –0.182, P = 0.043, 95% CI (–0.382, –0.006)] and consummatory anhedonia (TEPS-C) [B = –0.228, SE = 0.094, β = –0.214, P = 0.016, 95% CI (–0.413, –0.042)] were independently and negatively associated with PANSI. NSSI was also independently associated with PANSI [B = 0.092, SE = 0.026, β = 0.230, P < 0.001, 95% CI (0.042, 0.143)]. Notably, depressive symptoms (HAMD) did not remain significantly associated in the full model (P = 0.319). The remaining variables did not reach significance (all P > 0.05). All VIF values were below 2.5. In summary, after adjustment, anticipatory (TEPS-A) and consummatory anhedonia (TEPS-C) remained significantly associated with suicidal ideation (PANSI), whereas depressive symptoms did not.

Associations with Non-Suicidal Self-Injury (NSSI): A hierarchical multiple linear regression was conducted with NSSI severity as the dependent variable. In Block 1, diagnosis subtype, and anxiety symptoms (HAMA) were entered as covariates. In Block 2, depressive symptoms (HAMD), four dimensions of anhedonia (TEPS-A, TEPS-C, RSAS, RPAS), and suicidal ideation (PANSI) were simultaneously entered. Block 1 accounted for 3.2% of the variance [R² = 0.032, adjusted R² = 0.020, F(3, 239) = 2.650, P = 0.050]. The addition of Block 2 significantly improved the model, explaining an additional 19.6% of variance [ΔR² = 0.196, ΔF(6, 233) = 9.848, P < 0.001]. In the full model, after controlling for diagnosis subtype, and anxiety symptoms, somatic anhedonia (RPAS) [B = 0.642, SE = 0.197, β = 0.287, P = 0.001, 95% CI (0.255, 1.030)] and suicidal ideation (PANSI) [B = 0.576, SE = 0.159, β = 0.231, P < 0.001, 95% CI (0.263, 0.890)] were independently and positively associated with NSSI. Notably, depressive symptoms (HAMD) did not remain significantly associated in the full model (P = 0.665). The remaining variables did not reach significance (all P > 0.05). All VIF values were below 2.5. In summary, somatic anhedonia (RPAS) and suicidal ideation (PANSI) remained significantly associated with NSSI after covariate adjustment.

Associations with Suicide Attempt (SA): A hierarchical binary logistic regression was conducted with Suicide Attempt (SA, yes or no) as the dependent variable. In Block 1, diagnosis subtype, and anxiety symptoms (HAMA) were entered as covariates. In Block 2, depressive symptoms (HAMD), four dimensions of anhedonia (TEPS-A, TEPS-C, RSAS, RPAS), and suicidal ideation (PANSI), Non-Suicidal Self-Injury (NSSI) were simultaneously entered. Block 1 yielded a Nagelkerke R² = 0.030 and –2 log-likelihood = 317.059. The addition of Block 2 significantly improved model fit (Δ–2LL = 69.183, P < 0.001; –2 log-likelihood =247.186, Nagelkerke R² increased to 0.360). In the full model, after controlling for diagnosis subtype, and anxiety symptoms, somatic anhedonia (RPAS) [OR = 1.050, B = 0.049, P = 0.024, 95% CI (1.006, 1.097)], suicidal ideation (PANSI) [OR = 1.050, B = 0.049, P = 0.005, 95% CI (1.015, 1.087)], and non-suicidal self-injury (NSSI) [OR = 1.032, B = 0.031, P < 0.001, 95% CI (1.017, 1.047)] were significantly and independently associated with SA. Notably, depressive symptoms (HAMD) were not significant in the full model (P = 0.574). The remaining variables did not reach significance (all P > 0.05). In summary, somatic anhedonia (RPAS), suicidal ideation (PANSI) and NSSI remained significantly associated with suicide attempts after adjustment.

### 3.4 Exploratory mediation models analysis results

#### 3.4.1 Indirect statistical associations involving TEPS-A and TEPS-C in the relationship between HAMD and PANSI

With HAMD as the independent variable, PANSI as the dependent variable, and the TEPS-A, TEPS-C as the mediator. Exploratory mediation models (Process Model 4) effect testing indicated that the indirect effect through TEPS-A was statistically significant [B = 0.051, SE = 0.026, 95% CI (0.006, 0.105)], accounting for 20.98% of the total effect. By contrast, the indirect effect through TEPS-C did not reach statistical significance [B = 0.032, SE = 0.022, 95% CI (−0.001, 0.084)]. These results indicate that the statistical association between depressive symptoms and suicidal ideation was partially accounted for by individual differences in anticipatory anhedonia. (Details are shown in **Table 3**).

**Table 3:** Indirect statistical associations involving TEPS-A and TEPS-C in the Relationship Between HAMD and PANSI.

Type	B	SE	95% CI		Percentage of effect
			LLCL	ULCL	
total effect	0.243	0.069	0.107	0.378	
direct effect	0.160	0.066	0.030	0.290	65.88%
indirect effect					
total indirect effect	0.083	0.027	0.036	0.142	34.12%
TEPS-A	0.051	0.026	0.006	0.105	20.98%
TEPS-C	0.032	0.022	-0.001	0.084	13.14%
TEPS-A vs TEPS-C	0.019	0.039	-0.062	0.094	

#### 3.4.2 Indirect statistical associations involving RPAS in the relationship between HAMD and NSSI

With HAMD as the independent variable, NSSI as the dependent variable, and the RPAS as the mediator. Exploratory indirect effect model (Process Model 4) effect testing indicated that the total effect of HAMD on NSSI was statistically significant [B = 0.487, SE = 0.172 95% CI (0.005, 0.148)], while the direct effect was not (B = 0.247, SE = 0.165 95% CI [-0.077, 0.570]). RPAS showed a significant indirect effect [B = 0.241, SE = 0.070, 95% CI (0.118, 0.389)], accounting for 49.40% of the total effect. (Details are shown in **Table 4**).

**Table 4:** Indirect statistical associations involving RPAS in the relationship between HAMD and NSSI.

Type	B	SE	95% CI		Percentage of effect
			LLCL	ULCL	
total effect	0.487	0.172	0.005	0.148	
direct effect	0.247	0.165	-0.077	0.570	
indirect effect	0.241	0.070	0.118	0.389	49.40%

#### 3.4.3 Exploratory sequential indirect effects of RPAS, PANSI, NSSI in the relationship between HAMD and SA(suicide attempts)

With HAMD as the independent variable, SA as the dependent variable, and RPAS,PANSI,NSSI as mediating variables, exploratory sequential model (Process Model 6) was constructed to test the significance of each specific indirect effect and contrast using the Bootstrap method (number of samples = 5,000, 95% confidence interval). The total indirect effect was statistically significant [B = 0.0403, SE = 0.0107, 95% CI (0.0228, 0.0654)], whereas the direct effect was not [B = -0.0089, SE = 0.0166, 95% CI (-0.0414, 0.0255)]. These results indicate that the statistical covariation between depressive symptoms and suicide attempt status was fully accounted for by the set of mediators. Among the specific indirect effects, the path via RPAS alone [Ind1: B = 0.0127, 95% CI (0.0034, 0.0256)] and the chain path via RPAS and NSSI (Ind4: B = 0.0080, 95% CI [0.0034, 0.0155]) reached statistical significance. The longest sequential indirect path via RPAS → NSSI → PANSI [Ind7: B = 0.0012, 95% CI (0.0002, 0.0026)] was also significant, though of small magnitude. (Details are shown in **Table 5**).

**Table 5:** Exploratory sequential indirect effects of RPAS,PANSI,and NSSI in the relationship between HAMD and SA.

Type		B	SE	95% CI	
				LLCL	ULCL
direct effect		-0.0089	0.0166	-0.0414	0.0255
indirect effect					
total indirect effect		0.0403	0.0107	0.0228	0.0654
Ind1	HAMD→RPAS→SA	0.0127	0.0056	0.0034	0.0256
Ind2	HAMD→NSSI→SA	0.0080	0.0053	-0.0003	0.0206
Ind3	HAMD→PANSI→SA	0.0071	0.0047	0.0005	0.0189
Ind4	HAMD→RPAS→NSSI→SA	0.0080	0.0031	0.0034	0.0155
Ind5	HAMD→RPAS→PANSI→SA	0.0017	0.0013	0.0001	0.0049
Ind6	HAMD→NSSI→PANSI→SA	0.0012	0.0009	-0.0001	0.0034
Ind7	HAMD→RPAS→NSSI→PANSI→SA	0.0012	0.0006	0.0002	0.0026
compare					
C1	Ind1 vs. Ind2	0.0043	0.0080	-0.0120	0.0200
C2	Ind1 vs. Ind3	0.0055	0.0081	-0.0107	0.0212
C3	Ind1 vs. Ind4	0.0046	0.0058	-0.0065	0.0167
C4	Ind1 vs. Ind5	0.0110	0.0057	0.0016	0.0237
C5	Ind1 vs. Ind6	0.0114	0.0057	0.0020	0.0245
C6	Ind1 vs. Ind7	0.0115	0.0056	0.0023	0.0244
C7	Ind2 vs. Ind3	0.0013	0.0071	-0.0131	0.0152
C8	Ind2 vs. Ind4	0.0004	0.0054	-0.0104	0.0116
C9	Ind2 vs. Ind5	0.0067	0.0053	-0.0023	0.0189
C10	Ind2 vs. Ind6	0.0072	0.0048	-0.0003	0.0185
C11	Ind2 vs. Ind7	0.0072	0.0054	-0.0016	0.0195
C12	Ind3 vs. Ind4	-0.0009	0.0058	-0.0117	0.0118
C13	Ind3 vs. Ind5	0.0055	0.0046	-0.0011	0.0166
C14	Ind3 vs. Ind6	0.0059	0.0046	-0.0003	0.0173
C15	Ind3 vs. Ind7	0.0060	0.0046	-0.0001	0.0172
C16	Ind4 vs. Ind5	0.0064	0.0030	0.0019	0.0134
C17	Ind4 vs. Ind6	0.0068	0.0033	0.0017	0.0145
C18	Ind4 vs. Ind7	0.0068	0.0030	0.0025	0.0141
C19	Ind5 vs. Ind6	0.0004	0.0014	-0.0019	0.0037
C20	Ind5 vs. Ind7	0.0005	0.0011	-0.0013	0.0034
C21	Ind6 vs. Ind7	0.0001	0.0008	-0.0016	0.0018

## 4 Discussion

This study found significant differences among different suicide risk groups of adolescent patients with mood disorders in various dimensions of anhedonia, suicidal ideation, and non-suicidal self-injury (NSSI) levels, showing a clear gradient pattern: high suicide risk group > low suicide risk group > healthy control group. This is consistent with previous research findings that patients with suicide attempts often have more severe anhedonia (29), and anhedonia is associated with self-injury behaviors (21). The correlation analysis results of this study suggest that depressive symptoms are significantly associated with the occurrence of suicidal ideation and self-injury behaviors. However, in the hierarchical regression analysis, depressive symptoms were not risk factors for suicidal ideation or self-injurious behaviors, suggesting that depressive symptoms do not directly account for variance in suicidal ideation or behaviors, but may show statistical associations via some other potential variables. This pattern was further explored in the exploratory mediation models analysis results. The results indicated that the statistical association between depressive symptoms and suicidal ideation/behavior was partially accounted for by anhedonia dimensions, consistent with hypothesized mechanistic pathways. Anhedonia is significantly associated with self-harm and is an independent risk factor for suicidal thoughts and behaviors (20, 30). A meta-analysis by Ducasse and others (31) indicates that anhedonia is independently associated with suicidal ideation, regardless of the severity of depressive symptoms. Ding’s national multi-center prospective cohort study found that anhedonia is a core risk factor for suicidal behavior (32).

This study found that different dimensions of anhedonia have significantly different patterns of association with self-harm in adolescent patients with mood disorders. This finding supports the multidimensional structure theory of anhedonia (33), suggesting that different dimensions of anhedonia may participate in the construction of suicide through different neuropsychological mechanisms (34). In this study, the Time Experience Pleasure Scale (TEPS), serving as a comprehensive measure of anticipatory and consummatory anhedonia, independently associated with suicidal ideation.; Both anticipatory and consummatory anhedonia were negatively correlated with suicidal ideation. However, Parallel mediation analysis suggested that anticipatory anhedonia showed a significant indirect effect in the statistical model linking depressive symptom and suicidal ideation, whereas the consummatory anhedonia does not reach statistical significance, that is, the statistical association involving anticipatory anhedonia is more prominent. This is consistent with previous research findings. Huerta (16) found that trait anticipatory reward is negatively correlated with self-injurious thoughts and self-injury frequency, that is, the more severe the deficit in anticipatory anhedonia, the higher the frequency of suicidal thoughts and plans. Research by Rajesh (34) suggests that anticipatory anhedonia has stronger connections with “low mood” and “suicidal ideation” nodes in depressive symptoms, while consummatory anhedonia is associated with broader depressive symptoms (including feelings of worthlessness, sleep problems. Increasing research (35) indicates that anticipatory anhedonia and consummatory anhedonia have different neural mechanisms. Anticipatory anhedonia is closely related to abnormalities in the reward anticipation network, consistent with individuals’ reduced ability to anticipate future rewards. Consummatory anhedonia is more associated with the sensorimotor cortex and default mode network, not significantly related to classical reward anticipation circuits. Gupta (36) found that the level of neural activation in the ventromedial prefrontal cortex modulates the relationship between anhedonia and suicidal ideation. When it is highly activated in response to rewards, all types of anhedonia (anticipatory, consummatory, social) are associated with stronger suicidal ideation. Adolescents with depression show reduced activation in the striatum and medial prefrontal cortex during the reward anticipation stage. This deficit in reward processing may lead to a decreased ability to anticipate future positive experiences, preventing them from generating more motivation to plan for rewards, which in turn leads to feelings of hopelessness and suicidal thoughts (29). These findings are consistent with the hypothesis that interventions targeting anticipatory anhedonia might warrant investigation in relation to suicidal thoughts, though causal efficacy cannot be determined from cross-sectional data.

On the other hand, this study found that somatic anhedonia (RPAS) independently associated with non-suicidal self-injury and suicide attempts. Regression analysis indicated that somatic anhedonia is independently associated with non-suicidal self-injury and suicide attempts. Further mediation models suggested that the path via RPAS is dominant: the simple mediation path HAMD→RPAS→SA (B = 0.0127) is the single path with the strongest effect, and the sequential indirect path HAMD→RPAS→NSSI→SA (B = 0.0080) is the sequential path with the strongest effect. Notably, the path solely via NSSI (Ind2) is not significant, but it becomes significant when NSSI is combined with RPAS (Ind4) or RPAS and PANSI (Ind7), suggesting that RPAS emerged as a central variable in this statistical mediation pattern. This pattern is consistent with previous research, indicating that somatic anhedonia may be related to NSSI through a ‘sensation-seeking’ mechanism. Some NSSI patients engage in self-injury to generate physical sensations to counteract emotional numbness, a compensatory mechanism formed in response to the ‘inability to feel’ caused by somatic anhedonia (37). At the same time, somatic anhedonia is associated with non-suicidal self-injury (NSSI) behavior, possibly mediated through pain perception mechanisms. Koenig and colleagues (38) found that adolescents with NSSI exhibited significantly higher pain thresholds and increased pain tolerance under cold pain stimulation, that is, a reduced perception of pain. NSSI patients show significantly lower activation in the insula and ACC during pain stimulation compared to healthy controls, suggesting dulled pain perception (39). The loss of physical pleasure further increases pain tolerance and reduces behavioral inhibition toward self-harm. Studies (40) also indicate that NSSI behaviors can produce immediate emotional relief by activating the endogenous opioid system, forming a negative reinforcement cycle. Individuals with severe loss of physical pleasure experience changes in sensitivity to physical pain and are more likely to use NSSI as a means of emotion regulation. The FAST-MAS trial by Pizzagalli (41) further confirmed that κ-opioid receptor antagonists can significantly improve anhedonic behavior, suggesting that the opioid system is a therapeutic target. Therefore, somatic anhedonia may increase suicide risk through multiple mechanisms: (1) blunted pain perception, which increases pain tolerance and reduces inhibition against self-injury or suicidal behavior. (2) emotional relief effects, forming a negative reinforcement cycle that increases susceptibility to extreme sensation-seeking.

The sequential indirect effect model in this study identified potential associative patterns linking depressive symptoms to suicidal behavior, though these statistical patterns should not be interpreted as evidence of causal or temporal sequencing given the cross-sectional design. The core finding was that somatic anhedonia (RPAS) is a key hub statistically associated with both depressive symptoms and suicidal behaviors. First, the dominant statistical role of RPAS. Whether in simple path (Ind1) or sequential indirect path (Ind4, Ind5, Ind7), the pathways through RPAS were statistically significant, while the pathway through NSSI alone (Ind2) was not. This pattern is consistent with the theoretical view that “somatic anhedonia is strongly associated with suicidal behavior”. Second, the indirect effect involving suicidal ideation was smaller in magnitude, a pattern consistent with the hypothesis that depression may be linked to NSSI through cognitive associations (hopelessness, desire for death), not limited to affect regulation mechanisms. Zielinski et al. (37), which found that anhedonia can predict the perceived usefulness of NSSI but cannot directly predict NSSI frequency. By including suicidal ideation as a mediator in the model, this study explored a hypothesized the cognitive-motivational association linking depressive symptoms to NSSI complementing the sensory-physiological pathway represented by somatic anhedonia. This study included both somatic and suicidal ideation, which may have enabled the detection of a stronger mediating effect. Importantly, given that all variables were measured concurrently, any reference to temporal ordering below reflects theoretical speculation rather than empirical demonstration of temporal precedence. The observed pattern is consistent with a hypothesized temporal sequence in which somatic anhedonia precedes suicidal thoughts. Specifically, we speculate that somatic anhedonia may temporally precede suicidal ideation and could be indirectly associated with suicide risk by its co-occurrence with suicidal ideation. This hypothesis aligns with neurodevelopmental and clinical observations: somatosensory processing involves more primitive neural circuits (such as thalamo-cortical pathways) (42), whereas the formation of suicidal ideation requires higher-level cognitive evaluations (such as feelings of hopelessness about the future or perceived burden on the self) (43). if somatic anhedonia persists, individuals might gradually develop cognitions like ‘life is meaningless’ and ‘the future is hopeless,’ which could co-occur with suicidal ideation and NSSI behaviors as a way to cope with this psychological pain.Third, the longest sequential indirect path found in this study (HAMD→RPAS→NSSI→PANSI→SA, Ind7), although had the smallest effect size (B = 0.0012), was still statistically significant, a finding consistent with the Gradual Escalation Theory (44). This theory suggests that suicidal behavior does not occur suddenly, but gradually escalates through precursor behaviors such as NSSI. NSSI and suicidal behaviors are not entirely separate phenomena, but different functional expressions existing on the same multidimensional continuous spectrum.Within a theoretical model, depressive symptoms are hypothesized to precede somatic anhedonia,which, is posited to precede NSSI behavior in search of emotional regulation. Consistent with this theory, when NSSI fails to relieve the pain, suicidal thoughts may emerge. Longitudinal studies (45) show that when patients with NSSI revisit the clinic, most do not repeat NSSI, but exhibit suicidal ideation and suicide attempts, supporting the theory of progression from NSSI to suicidal behavior. At the same time, the transition from suicidal ideation to action requires “acquiring the capability for suicide” (46, 47). Once an individual has suicidal thoughts, repeated self-injury may occur, and through habituation to physical pain and fear responses, the inhibition toward suicidal behavior decreases, increasing the risk of suicide (48), thus creating a vicious cycle. This process appears to coincide with an interesting finding of this study: non-suicidal self-injury and suicidal ideation showed strong mutual associations, both of which were associated with the occurrence of suicidal behavior. Again, these co-occurrence patterns cannot establish directional or causal relationships.

This study found that social anhedonia does not have a significant predictive effect on suicidal ideation, NSSI, or suicide attempts,which differs from previous results. Possible reasons include the diversity of measurement tools for social anhedonia, which may lead to inconsistent research results. This study used the Chapman Revised Social Anhedonia Scale (RSAS) to assess trait social anhedonia, which focuses on stable personality traits rather than changes in state social pleasure experiences. However, recent studies (49) have shown that state social anhedonia is directly related to suicidal ideation, while trait social anhedonia only indirectly influences suicidal ideation by moderating the relationship between interpersonal distress and suicidal thoughts. Other research (50) indicates that social anhedonia indirectly affects suicide risk through loneliness; loneliness, as an important driver of suicide, increases suicide risk, whereas social anhedonia is not a direct risk factor for suicide.

This study has several limitations. First, the cross-sectional design precludes causal inference and temporal ordering; all indirect effects represent statistical associations rather than evidence of causal pathways, and reverse causality remains plausible. Future research should adopt longitudinal designs to track the developmental trajectories of dimensions of anhedonia, NSSI, and suicidal behavior. Second, Our sample was predominantly composed of MDD patients (83.3%), with bipolar disorders accounting for only 16.7%. This imbalance limits the generalizability of our findings to bipolar youth, who may exhibit distinct neurobiological and clinical profiles regarding anhedonia and self-harm.Future studies should recruit larger, diagnostically balanced samples to examine whether the observed dimensional-specific patterns differ across mood disorder subtypes. Third, the data of suicide-risk were collected through use of HAMD item 3 rather than structured specific suicide-risk instrument. Thus, the severity of suicide-risk assessed is not sufficiently systematic and reliable. Therefore, replications based on structured specific suicide-risk instrument are also needed. Forth we did not assess borderline personality traits or childhood trauma experiences, both of which have been consistently implicated in the pathophysiology of mood disorders and are known to influence self-harm. Future studies should incorporate comprehensive assessments of borderline personality traits and childhood trauma histories to better examine potential interactions between anhedonia, borderline traits, and early adversities in shaping self-harm outcomes. Self-report measures, while feasible, may be influenced by social desirability and recall bias. Future studies could incorporate behavioral tasks (e.g. monetary incentive delay tasks) and physiological indicators (e.g. skin conductance response) for multi-method assessments.

In summary, this study demonstrated that different dimensions of anhedonia are differently associated with self-harm indicators: anticipatory anhedonia was statistically associated with suicidal ideation. In contrast, somatic anhedonia (RPAS) was associated with non-suicidal self-injury (NSSI) and suicidal attempts. This supported the theoretical framework of anhedonia as a multidimensional construct and suggests that clinical assessments and interventions should differentiate between dimensions of anhedonia. These findings suggest that clinical assessment might benefit from differentiating anhedonia dimensions. Patients reporting suicidal ideation showed distinct anhedonia profiles compared to those reporting NSSI or suicide attempts, raising the hypothesis that targeted interventions focused on anticipatory pleasure versus somatic anhedonia may warrant investigation in future experimental or longitudinal studies.

## Data Availability

The raw data supporting the conclusions of this article will be made available by the authors, without undue reservation.
